# Immune monitoring, immune phenotyping, and precision intervention in severe pneumonia: current evidence and translational challenges

**DOI:** 10.3389/fimmu.2026.1898737

**Published:** 2026-09-10

**Authors:** Fangfei Ren, Jun Gao, Xuan Zhao, Qiongling Sun, Kun Tang, Tianyi Wang, Wenjie Guo, Qi Pan, Zikun Fang, Wensen Pan, Jing Yu

**Affiliations:** Second Department of Respiratory and Critical Care Medicine, The Second Hospital of Hebei Medical University, Shijiazhuang, China

**Keywords:** immune dysregulation, immune monitoring, immune phenotyping, precision medicine, severe pneumonia

## Abstract

Severe pneumonia remains a major cause of mortality in critically ill patients, with severe community-acquired pneumonia representing a clinically important and representative scenario. This review focuses primarily on severe community-acquired pneumonia as the clinical context, in which outcomes are determined by the interplay between pathogen burden and heterogeneity of host responses. Beyond pathogen-directed therapies, severe pneumonia is frequently characterized by dynamic and partially overlapping states of hyperinflammation, immune dysfunction, and subsequent immunosuppression. Different host conditions, including immunocompromised states, advanced age, post-sepsis critical illness, and pediatric populations, further increase the complexity and heterogeneity of immune responses. These immune states can be evaluated through conventional inflammatory biomarkers, cellular immune parameters, cytokine profiling, and emerging high-dimensional profiling technologies. This review summarizes the immunopathological basis of severe pneumonia and presents an integrated translational framework encompassing immune monitoring, immune phenotype characterization, risk stratification, evidence-based intervention selection, and serial reassessment. Importantly, immune phenotypic classification should be distinguished from prognostic prediction: models capable of predicting mortality risk do not necessarily identify biologically meaningful immune phenotypes that are amenable to therapeutic intervention. Current evidence supports the use of immune biomarkers primarily as complementary tools for risk stratification and hypothesis-driven patient selection, whereas most immune-targeted interventions remain investigational. Future clinical implementation requires standardized assays, phenotype definitions that incorporate both pathogen characteristics and disease trajectory, external validation, and prospective clinical trials demonstrating improvements in clinically meaningful patient outcomes.

## Graphical Abstract

**Figure:**
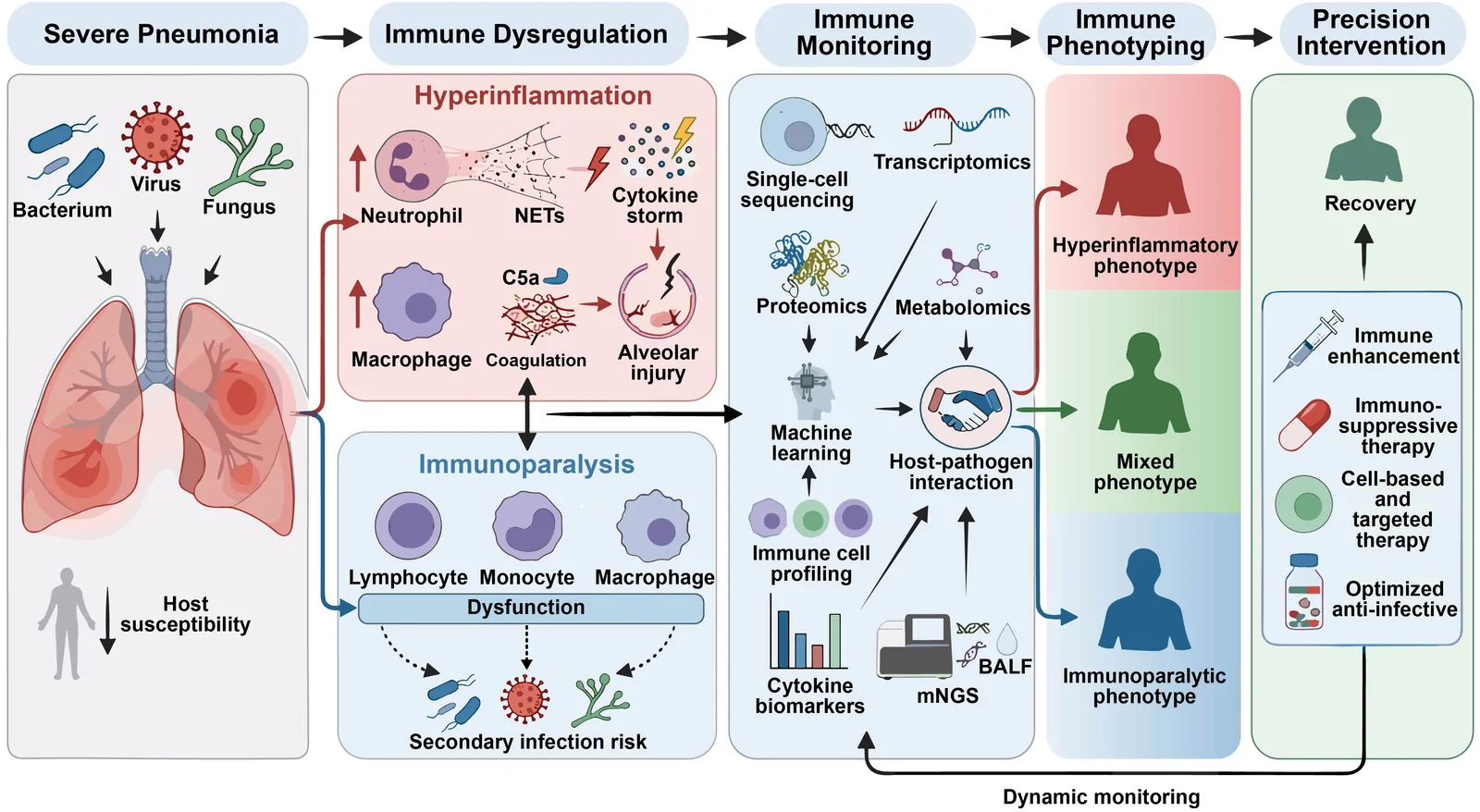
Immune monitoring-guided precision management in severe pneumonia. *Note:* Host factors, pathogen characteristics, and disease stage jointly shape immune responses in severe pneumonia. A stepwise framework integrates immune biomarkers, pathogen assessment, and advanced profiling technologies to identify hyperinflammatory, immunoparalyzed, and mixed or dynamically evolving immune states. These phenotypes may support individualized anti-infective optimization and phenotype-informed immunomodulatory strategies. Serial reassessment of immune and pathogen status enables treatment adjustment and establishes a closed-loop precision management model. The arrows indicate an iterative clinical loop rather than a linear one-time decision process. NETs, Neutrophil extracellular traps; mNGS, metagenomic next-generation sequencing; BALF, bronchoalveolar lavage fluid.

## 1 Introduction

The incidence and mortality of severe pneumonia remain persistently high, particularly in the intensive care unit settings. Among patients with severe pneumonia, severe community-acquired pneumonia (sCAP) represents the most common and extensively studied clinical phenotype, serving as the principal foundation for current research on precision assessment and management of severe pneumonia. Despite substantial advances in antimicrobial therapy and supportive care, clinical improvements remain limited and outcomes remain poor, highlighting the limitations of conventional management strategies that primarily focus on pathogen identification and empirical pathogen-directed treatment (1). Increasing evidence suggests that the trajectory and clinical outcomes of pneumonia are determined not only by the pathogen itself, but more importantly by the magnitude, direction, and dynamic evolution of the host immune response (2). Notably, host immune responses in patients with severe pneumonia exhibit marked heterogeneity: some patients fail to effectively clear pathogens due to insufficient immune responses, whereas others develop severe tissue injury and even multiple organ dysfunction syndrome (MODS) as a consequence of excessive inflammatory responses (3, 4). The coronavirus disease 2019 (COVID-19) pandemic has further reinforced this concept, underscoring the central role of host immune dysregulation in the pathogenesis and progression of severe pneumonia, and suggesting that a “one-size-fits-all” anti-infective strategy is inadequate to meet the diverse clinical needs of different patient populations (5, 6).

Severe pneumonia is not a single disease entity, but rather a heterogeneous clinical syndrome of severe pulmonary infection determined by the interplay of different pathogens, infection sources, and host-related factors. It is characterized by severe respiratory dysfunction, hemodynamic instability, and/or extrapulmonary organ dysfunction, and may require intensive care and organ support therapies (7). Currently, different types of severe pneumonia are assessed using distinct severity criteria according to their infection sources and clinical contexts. Various forms of severe pneumonia, including sCAP, hospital-acquired pneumonia (HAP), ventilator-associated pneumonia (VAP), and severe virus-associated pneumonia, differ substantially in pathogen profiles, host immune status, and mechanisms underlying disease progression. Therefore, this review primarily uses sCAP as the clinical model and integrates evidence from other types of severe pneumonia to explore both the shared and distinct features of immune dysregulation, immune monitoring, and precision intervention strategies across different clinical settings. sCAP currently represents the most extensively investigated clinical model in severe pneumonia research, with disease severity commonly assessed according to the IDSA/ATS criteria, which define severe pneumonia as the presence of at least one major criterion or at least three minor criteria. The major criteria include the requirement for invasive mechanical ventilation or septic shock requiring vasopressor support (8).

With the growing understanding of host immune responses, current concepts in pneumonia research and management are shifting from a “pathogen-centered” paradigm toward a “host–pathogen interaction–oriented” framework (9). This transition implies that clinical decision-making should not only focus on etiological diagnosis and antimicrobial selection but also emphasize the identification and stratification of the host immune status. In this review, immune phenotype refers to an observable pattern of immune characteristics defined by measurable immune features at a specific stage of disease, reflecting the current immune response profile of a patient rather than a fixed or immutable biological subtype. For instance, patients exhibiting prominent immunosuppressive features may potentially benefit from immune-enhancing strategies, whereas those with pronounced hyperinflammatory profiles may be more suitable candidates for anti-inflammatory modulation. However, therapeutic decisions should be guided by dynamic assessments of the overall immune status (3). Nevertheless, rapid, standardized, and reproducible tools for immune monitoring are still lacking in current clinical practice, thereby limiting the implementation of precision immunomodulatory strategies. Although previous reviews have summarized immune abnormalities and precision therapeutic strategies in severe pneumonia from the perspectives of immunopathological mechanisms, inflammatory dysregulation, and specific immune-modulatory interventions, a systematic synthesis of the continuous translational pathway encompassing “immune monitoring, immune phenotype characterization, risk stratification, evidence-based intervention selection, and serial reassessment” remains lacking. In particular, comprehensive evaluations are still insufficient regarding the dynamic evolution of immune states across different host backgrounds, the clinical feasibility of immune monitoring technologies, the validation status of biomarkers, and the maturity of evidence supporting immune phenotype-guided therapeutic strategies. Therefore, this review primarily focuses on sCAP as a representative clinical model, while incorporating evidence from other severe pneumonia settings, to systematically examine the immunopathological mechanisms of severe pneumonia, immune landscapes in special populations, immune monitoring technologies, immune phenotypes, and precision intervention strategies. The aim of this review is to summarize current advances, evaluate the strength and limitations of existing evidence, and discuss the future prospects and challenges of immune-guided precision therapy in clinical practice.

## 2 Literature search strategy

This review was conducted as a narrative review to provide a comprehensive overview of immune dysregulation, immune monitoring, immune phenotypes, and precision intervention strategies in severe pneumonia. Literature searches were performed in PubMed and Web of Science, covering the period from database inception to February 2026. The search strategy included combinations of the following keywords: “severe pneumonia”, “severe community-acquired pneumonia”, “hospital-acquired pneumonia”, “ventilator-associated pneumonia”, “COVID-19 pneumonia”, “acute respiratory distress syndrome”, “sepsis”, “host response”, “immune dysregulation”, “immune monitoring”, “immune phenotype”, “immune endotype”, “biomarker”, “multi-omics”, “single-cell sequencing”, “immunomodulatory therapy”, and “precision medicine”. Search terms were adapted as appropriate for each database.

Studies were selected primarily according to their relevance to the scope of this review, including: (1) basic and translational studies investigating host immune responses and immunopathological mechanisms in severe pneumonia; (2) clinical studies evaluating immune cells, inflammatory mediators, molecular biomarkers, and omics-based technologies for disease stratification or prognostic assessment; and (3) clinical trials, cohort studies, and case reports examining immune phenotype-guided therapeutic strategies. Animal and *in vitro* studies were included only when they provided clear mechanistic insights relevant to clinical immunomodulatory approaches. Differences in the level of evidence across study designs are considered and discussed throughout the review. No language restrictions were applied during the literature search; however, priority was given to peer-reviewed studies published in English. Conference abstracts, non-peer-reviewed publications, and sources for which the full text was unavailable were not included.

## 3 Multidimensional dysregulation of immunopathological mechanisms

Quantitative assessment of host immune responses in severe pneumonia fundamentally relies on an integrated understanding of its complex and dynamic immunopathological mechanisms. These immunopathological alterations not only constitute the biological basis underlying immune heterogeneity in severe pneumonia but also provide a biological foundation for subsequent immune monitoring, biomarker discovery, and immune phenotype-based stratification. This process is essentially characterized by spatiotemporal dysregulation between innate and adaptive immunity, featuring the coexistence of excessive local pulmonary inflammation and systemic immunosuppression, which may ultimately progress to MODS. Accordingly, multiple dimensions—including immune cell functional states, inflammatory mediator networks, and inter-organ crosstalk—constitute critical pathological domains that can be quantitatively evaluated.

### 3.1 Innate immune dysregulation and inflammatory amplification

At the level of innate immunity, neutrophils and alveolar macrophages (AMs) serve as the principal effector cells in the early anti-infective response, with their recruitment, activation, and functional states being particularly critical. Under physiological conditions, these two cell populations coordinate pathogen clearance, inflammatory regulation, and tissue repair. However, in severe pneumonia, dysregulated neutrophil and AMs responses can lead to persistent amplification of inflammation and exacerbation of pulmonary tissue injury (10, 11). Among these alterations, neutrophil dysregulation represents a hallmark feature of innate immune imbalance in severe pneumonia. Single-cell transcriptomic analysis revealed that neutrophils are markedly expanded and dominate the inflammatory response in severe pneumonia caused by severe acute respiratory syndrome coronavirus 2 (SARS-CoV-2), highlighting their pivotal role in disease progression (12). However, neutrophil-mediated tissue injury is not solely determined by increased neutrophil abundance but is also closely associated with their effector functions following activation. Zhang et al. (13) further observed a significant increase in neutrophil extracellular traps (NETs) in critically ill patients, which sustain inflammatory amplification through the formation of positive feedback loops. Additional studies indicate a complex interplay between NETosis and inflammatory programmed cell death, whereby inflammasome activation and proinflammatory cytokine release exacerbate systemic inflammation and immune dysregulation (14). Notably, NETosis is not a unidirectional pro-injury process but is finely regulated by multiple molecular mechanisms. For instance, C-type lectin receptors can restrict NET formation, thereby attenuating inflammatory injury (15), whereas peptidylarginine deiminase 2-mediated, caspase-1-dependent cell death promotes inflammatory injury (16). These findings indicate that NETosis is tightly regulated by multiple molecular pathways, and distinct regulatory mechanisms can influence its dual roles in host antimicrobial defense and inflammatory injury. Beyond inflammatory amplification, neutrophil dysfunction can also directly impair host antimicrobial capacity. Furthermore, Sprenkeler et al. (17) reported that deficiency of megakaryoblastic leukemia 1 impairs neutrophil chemotaxis and disrupts cytoskeletal remodeling, thereby weakening their antimicrobial function. Meanwhile, studies in invasive aspergillosis have shown that neutrophils contribute to antifungal immune defense by regulating natural killer cells (NK cells) recruitment via chemokine signaling (18). Therefore, beyond alterations in cell abundance, functional states including neutrophil migration, NETosis, and intercellular interactions also critically shape host antimicrobial responses. In contrast, AMs play a more central regulatory role in modulating inflammation and maintaining pulmonary homeostasis (10). During the early phase of infection, AMs preserve pulmonary immune equilibrium through pathogen phagocytosis, antigen presentation, and modulation of inflammatory responses (19). However, functional impairment of AMs has been shown to directly facilitate viral dissemination and exacerbate pneumonia severity (20). Following resolution of inflammation, AMs may undergo epigenetic reprogramming, resulting in sustained impairment of phagocytic capacity and antigen-presenting function, thereby contributing to immunoparalysis and increased susceptibility to secondary infections (21). Furthermore, in VAP, AMs regulate ferroptosis in pulmonary epithelial cells through the AKT3/GPX4 signaling pathway, thereby contributing to lung tissue injury (22). These findings indicate that AMs participate not only in pathogen clearance but also in inflammatory regulation and tissue injury, with their functional states dynamically changing throughout disease progression. Overall, dysregulated functions of neutrophils and AMs jointly contribute to innate immune abnormalities in severe pneumonia. These abnormalities involve not only alterations in cellular abundance but also multiple functional processes, including recruitment, activation, and effector responses, thereby providing an important pathological basis for subsequent inflammatory amplification and tissue damage.

### 3.2 Adaptive immune remodeling and immune-mediated injury

Adaptive immune dysregulation represents an important mechanism underlying the persistent progression of severe pneumonia. Such abnormalities involve not only impaired immune function but also remodeling of immune cell subset composition and functional states (19). T-cell-mediated cellular immunity and B-cell-mediated humoral immunity jointly contribute to pathogen clearance, inflammatory regulation, and maintenance of immune homeostasis, and dysfunction of either component may influence disease outcomes. T-cell immune abnormalities in severe pneumonia are primarily characterized by dysregulated immune responses rather than simply enhanced immune activation. In patients with severe H1N1 influenza pneumonia, the proportions of peripheral blood Th2 and Tc2 cells were significantly increased, accompanied by persistent elevation of pro-inflammatory cytokines, including interleukin (IL)-6, C-C motif chemokine ligand 3 (CCL3), and C-X-C motif chemokine ligand 8 (CXCL8). These findings suggest that T-cell subset polarization and inflammatory amplification jointly contribute to disease progression (23). Meanwhile, animal studies further demonstrated that pre-existing immune responses induced by SARS-CoV nucleocapsid protein prior to infection could result in enhanced Th1- and Th2-type cytokine responses upon subsequent infection, accompanied by more severe pulmonary immunopathological injury (24). Collectively, these studies indicate that the balance of T-cell responses, rather than the absolute magnitude of immune activation alone, more critically determines host immune responses and pulmonary tissue injury. In contrast, B-cell-mediated humoral immunity primarily influences the capacity for sustained pathogen clearance. Wei et al. (25) compared the clinical courses of SARS-CoV-2 infection in patients with two distinct forms of immunodeficiency. Patients with multiple myeloma receiving B-cell maturation antigen-targeted chimeric antigen receptor T-cell therapy exhibited persistent inability to clear the virus due to profound humoral immune deficiency and eventually died from cytokine storm and respiratory failure. In contrast, kidney transplant recipients receiving long-term cyclosporine treatment, despite impaired cellular immunity, were still able to generate virus-specific antibodies and ultimately achieve viral clearance. These findings suggest that different types of immunodeficiency may lead to distinct disease trajectories, and that humoral immune competence may serve as a critical determinant in specific populations with severe pneumonia.

### 3.3 Systemic inflammatory amplification and inter-organ crosstalk–mediated injury

The progression of localized pulmonary inflammation toward systemic inflammation represents a critical pathological process underlying the development of MODS in severe pneumonia (19). This process is not driven by a single inflammatory mediator but rather results from complex interactions among cytokine networks, the complement and coagulation systems, and interorgan immune signaling pathways. Dysregulation of cytokine networks constitutes an important basis for the persistent amplification of systemic inflammation. Multiple studies have demonstrated that IL-6 and IL-10 levels are closely associated with disease severity and clinical outcomes. Sustained elevation of IL-6 reflects excessive activation of inflammatory responses, whereas abnormal expression of anti-inflammatory cytokines such as IL-10 indicates impaired immune regulation. Together, these cytokines contribute to the inflammatory microenvironment characteristic of severe pneumonia (26–28). The inflammatory response further activates complement and coagulation cascades, forming a mutually reinforcing inflammatory amplification network. Müller-Redetzky et al. (29) reported significantly elevated serum C5a levels in patients with pneumonia, which contribute to lung–liver injury by increasing vascular permeability and recruiting inflammatory cells. Coagulation abnormalities are also commonly observed in severe pneumonia; even in non-critically ill patients, sustained elevations of D-dimer and thrombin–antithrombin complexes suggest a tight coupling between inflammation and coagulation activation (30). The aforementioned studies indicate that complement activation and coagulation abnormalities are not merely consequences of inflammatory responses but can also further amplify inflammatory injury. As inflammation continues to expand, localized pulmonary injury gradually evolves into dysregulation of interorgan immune networks. Reyes et al. (31) demonstrated that Streptococcus pneumoniae infection can directly induce myocardial injury, while studies on mechanical ventilation indicate that it may exacerbate systemic inflammation and promote the development of acute respiratory distress syndrome (ARDS) (32). In addition, bacterial virulence factors and molecular signaling pathways play critical roles in shaping the immune microenvironment. For example, Pseudomonas aeruginosa induces phagocyte dysfunction via Exoenzyme U (33), whereas antibodies targeting PcrV/Psl can attenuate inflammatory responses and confer protective effects (34); modulation of the triggering receptor expressed on myeloid cells-1 pathway has also been shown to improve infection outcomes (35). At the molecular level, hydrogen peroxide disrupts the alveolar barrier via inflammasome activation and endoplasmic reticulum stress (36), while lipopolysaccharide impairs endothelial barrier integrity by inhibiting Rac family small GTPase 1 activity (37). These findings suggest that pulmonary barrier disruption results not only from inflammatory cell infiltration but is also closely associated with dysregulated intracellular signaling pathways.

Collectively, multidimensional immune dysregulation constitutes the biological basis for implementing immune monitoring in severe pneumonia. Innate immune abnormalities, adaptive immune dysfunction, persistent immunosuppression, and inflammation-associated endothelial and coagulation injuries are not isolated processes; rather, they may coexist, alternate, or dynamically evolve across different stages of disease. Therefore, interpretation of their clinical significance requires comprehensive consideration of disease trajectory, pathogen-related factors, host characteristics, and therapeutic exposures. Accordingly, immune monitoring in severe pneumonia should not be viewed as reliance on a single laboratory parameter but rather as an integrated framework for capturing dynamic patterns of host responses.

## 4 Immune heterogeneity in special host populations

Building upon the multidimensional dysregulation of immunopathological mechanisms described above, heterogeneity among special populations—including immunocompromised hosts, elderly patients, post-sepsis individuals, and pediatric populations—further amplifies the complexity and diversity of the immune landscape. A comprehensive understanding of the immune characteristics of these populations not only helps explain their increased susceptibility to infection and poor clinical outcomes but also highlights that subsequent immune monitoring and immune phenotype-based stratification should be interpreted within the context of host-specific environments, providing an important foundation for advancing individualized precision interventions.

### 4.1 Immunodeficiency characteristics in immunocompromised hosts

Immunocompromised hosts constitute a highly heterogeneous population, in which the source and degree of immune deficiency directly influence the pathogen spectrum, inflammatory responses, and disease progression. Among solid organ transplant recipients, particularly kidney transplant patients, long-term T-cell-targeted immunosuppression therapy leads to persistent impairment of adaptive immunity, thereby reducing early pathogen clearance capacity. Cremoni et al. (38) demonstrated that in kidney transplant recipients with mild disease, reduced T-cell responsiveness following baseline polyclonal stimulation in the early phase of COVID-19 could predict subsequent progression to severe pneumonia. Zhuang et al. (39) further reported that alterations in the frequency and absolute counts of total regulatory T cells and their subsets are closely associated with pneumonia severity after kidney transplantation, with imbalance in specific subpopulations serving as potential indicators of disease progression risk. In patients with hematologic malignancies, immune deficiency presents with greater complexity. Zhou et al. (40) suggested that the high mortality of severe pneumonia following allogeneic hematopoietic stem cell transplantation may be related to lung injury mediated by the IL-6/sIL-6R/JAK1/STAT3 signaling pathway. Moreover, studies have shown that patients with multiple myeloma receiving daratumumab therapy have a high risk of cytomegalovirus (CMV) reactivation, thereby increasing the likelihood of secondary severe bacterial pneumonia (41, 42). These findings collectively suggest that different types of immunodeficiency not only determine susceptibility to infection but also shape distinct immunoinflammatory landscapes. Meanwhile, immunosuppressive states can further alter pathogen ecology. Metagenomic next-generation sequencing (mNGS) studies have demonstrated that the rate of mixed infections in immunocompromised patients with sCAP reaches 58.82%, with co-infections involving Pneumocystis jirovecii pneumonia (PJP), CMV, and various uncommon pathogens being more prevalent in this group (43–45). Notably, different types of immunosuppression are associated with distinct patterns of infection. For example, Kreitmann et al. (46) found that patients with hematological malignancies exhibited a lower 28-day cumulative incidence of ventilator-associated bacterial lower respiratory tract infections compared with other immunocompromised populations. This finding suggests that immunosuppression is not a uniform risk factor but rather encompasses distinct biological heterogeneity.

### 4.2 Immunosenescence and inflammaging in elderly patients

The immune characteristics of older adults are not simply characterized by impaired immune function but rather reflect a complex process of immune remodeling driven by the combined effects of immunosenescence and inflammaging. This host background places older individuals in a state of persistent low-grade chronic inflammation accompanied by declining immune competence. Consequently, immune responses following infection may be insufficient for rapid pathogen clearance while simultaneously being prone to developing sustained inflammatory activation (47, 48). This immune remodeling is first reflected by alterations in the regulation of inflammatory responses. Williams et al. (49) demonstrated in a Streptococcus pneumoniae infection model that aged mice exhibited enhanced pulmonary neutrophil recruitment, accompanied by reduced levels of the key anti-inflammatory cytokine IL-10 and increased expression of multiple chemokines, including CXCL9, CCL3, and CCL5. These findings suggest that older hosts may have impaired capacity to resolve inflammatory responses in a timely manner, resulting in sustained inflammatory amplification and exacerbated pulmonary tissue injury. In addition, Moreno Fernández-Ayala et al. (50) proposed that mitochondrial dysfunction may represent a critical mechanism underlying the exacerbation of cytokine storm and organ injury in infections such as COVID-19, particularly in the context of aging and its associated comorbidities, including metabolic syndrome and diabetes. Collectively, these studies indicate that the increased risk among older adults is attributable not only to impaired immune function but also to inflammatory dysregulation caused by reduced immune regulatory capacity. This age-associated immune landscape ultimately contributes to increased clinical susceptibility. In an analysis of patients admitted to intensive care units in France, Dananché et al. (51) found that although the overall incidence in elderly patients (≥75 years) is not necessarily significantly higher, the declining trend in risk is less pronounced, and the detection rate of multidrug-resistant pathogens is higher in this population. These findings suggest that age-related immune remodeling, underlying comorbidities, and long-term healthcare exposure collectively shape a unique infection risk profile in older adults.

### 4.3 Post-sepsis and critical illness–associated immunoparalysis

Patients in the post-sepsis state and those with chronic critical illness frequently develop persistent inflammation, immunosuppression, and catabolism syndrome (PICS). The defining feature of PICS is not complete resolution of inflammation, but rather the development of predominant acquired immune suppression and immunoparalysis under a background of persistent low-grade inflammation. One of the key characteristics of immuneparalysis is the prolonged inability of pulmonary innate immunity to regain normal function. Roquilly et al. (21) demonstrated that following resolution of pneumonia, the phagocytic capacity of AMs may remain impaired for several weeks, a phenomenon linked to epigenetic reprogramming and trained tolerance induced by local immunosuppressive signals; in critically ill patients, such reprogramming can persist in both AMs and circulating monocytes. These findings indicate that recovery from infection does not necessarily coincide with restoration of immune function, and persistent AM dysfunction may represent an important mechanism underlying long-term susceptibility to infection in critically ill patients. Furthermore, multiple iatrogenic factors during critical care management may further aggravate this immunoparalytic state. Bielen et al. (52) further reported that mechanical ventilation may reduce the phagocytic function of AMs by inducing pulmonary IL-4 secretion, thereby creating an immunosuppressive microenvironment conducive to the development of VAP. Meanwhile, at the level of the brain–lung–immune axis, neurocritical illness and traumatic brain injury can alter systemic cytokine expression profiles, thereby influencing the risk of VAP (53, 54). Viral infections can further amplify this process. In COVID-19-related ARDS, both the virus itself and immunomodulatory agents such as corticosteroids contribute to a pronounced state of immunoparalysis, leading to a high incidence of secondary VAP and opportunistic fungal infections (55–59). In recent years, researchers have attempted to dynamically evaluate this immunosuppressive state using biomarkers. Albert et al. (60) suggested that airway Candida colonization may reflect persistent inflammation and innate immune suppression, whereas Castain et al. (61) proposed that the dynamic viral load of non-enveloped viruses, such as torque teno virus, may serve as a potential biomarker for immunosuppression levels and the risk of HAP in critically ill patients. Therefore, immunoparalysis in post-sepsis and critically ill patients should not be considered a static condition but rather a dynamic state of immune dysregulation continuously shaped by the interplay among infection, organ injury, and critical care interventions.

### 4.4 Age-dependent immune characteristics in pediatric severe pneumonia

Pediatric patients exhibit distinct age-dependent immune characteristics in severe pneumonia due to the ongoing development of the immune system. This age-dependent pattern is first reflected in differences in local inflammatory responses. Severe pneumonia in infants is not simply characterized by insufficient immune activation; excessive activation of local pro-inflammatory pathways may also contribute to pulmonary tissue injury. For example, Liu et al. (62) reported that sCAP in infants younger than 1 year is closely associated with enhanced local Th17-driven inflammation, as evidenced by significantly elevated levels of IL-1β, IL-6, and IL-17 in bronchoalveolar lavage fluid (BALF). Further studies showed that overall changes in T-cell subsets and immunoglobulin levels were relatively limited among children with sCAP across different age groups; however, NK cells, complement component C4, and cytokines including IL-8 and IL-10 exhibited distinct age-related patterns, with higher levels observed in younger children (63). Therefore, immune heterogeneity in pediatric severe pneumonia is reflected not only by differences in inflammatory intensity but also by age-dependent alterations in innate immunity, cytokine regulation, and complement responses. In addition, immune states in pediatric patients are shaped by the combined effects of critical illness-related stress, nutritional status, and therapeutic interventions. Carcillo et al. (64) proposed that critical illness–acquired, stress-induced immunosuppression in children may represent a key mechanism underlying nosocomial infections, and is associated with deficiencies in micronutrients such as zinc, selenium, and glutamine. Therefore, unlike adults, the immune landscape of pediatric severe pneumonia is jointly shaped by developmental immune maturation, local inflammatory responses, and critical illness-associated immunosuppression. Accordingly, assessment and intervention strategies should incorporate immune developmental stage as a key variable.

Collectively, immune biomarkers should not be interpreted as fixed indicators independent of host context. The same biomarker may have different biological and clinical implications depending on age, immune developmental stage, baseline immunosuppressive exposure, transplantation status, organ dysfunction, and previous immunomodulatory therapies. Therefore, universal biomarker thresholds should be avoided unless they have been validated in the corresponding host populations. For special populations, longitudinal changes within individual patients, interpreted together with pathogen burden, treatment exposure, and clinical trajectories, may provide greater clinical value than isolated measurements. This host-context-guided approach may support personalized decisions regarding monitoring intensity, risk stratification, antimicrobial treatment duration, surveillance for secondary infections, and cautious selection of immunomodulatory strategies. However, prospective validation in specific populations remains necessary before such approaches can be incorporated into routine treatment allocation.

## 5 Key technological approaches for immune monitoring

Quantitative characterization of key immune cells and mediators involved in the immunopathology of severe pneumonia relies on advanced technological frameworks capable of high-dimensional and high-resolution dissection of host immune complexity. Immune monitoring encompasses not only the quantitative assessment of immune cell populations and functional states but also the comprehensive evaluation of inflammatory mediator levels, immune regulatory networks, and host response characteristics. Given the pronounced heterogeneity, dynamic nature, and network-like features of immune responses in severe pneumonia, traditional single-parameter assays may be insufficient to meet the demands of precise stratification. Therefore, the recent development of technologies such as flow cytometry, single-cell sequencing, and multi-omics approaches has provided new tools for characterizing immune cell composition, functional alterations, and trajectories of immune dysregulation. Meanwhile, pathogen detection based on mNGS and microbiome profiling can further complement the understanding of host–pathogen interactions, providing additional support for comprehensive assessment of disease progression and therapeutic responses. However, these technologies differ substantially in terms of the biological dimensions assessed, degree of standardization, clinical accessibility, and maturity of supporting evidence. Their value extends beyond mechanistic investigation and increasingly depends on whether they can generate reproducible, interpretable, and clinically actionable immune information. Therefore, this section reviews the characteristics, advantages, limitations, and clinical translation status of different immune monitoring technologies (**Table 1**).

**Table 1:** Characteristics and clinical maturity of immune monitoring technologies and host–pathogen integrated assessment approaches in severe pneumonia.

Technology	Quantifiable immune parameters	Sample requirements	Recommended timing of measurement	Turnaround time	Thresholds	Primary role	Major advantages	Limitations	Evidence level and clinical maturity
Flow cytometry	Lymphocyte subsets, mHLA-DR expression	Peripheral blood	Baseline assessment in critically ill patients; repeated measurements may be considered in cases of persistent organ dysfunction, secondary infection, or when immunomo- -dulatory therapy is under consideration	Several hours to 1 day	Influenced by analytical platforms, patient populations, and clinical settings; no universally accepted thresholds for treatment decision-making have been established.	Assessment of immune status; identification of immunoparalysis	Quantitative immune cell profiling at the cellular level	Results are influenced by sampling timing, analytical platforms, and processing procedures; thresholds vary across studies	Supported by multiple observational and translational studies; implemented in some specialized centers, but prospective validation for guiding immunotherapy remains limited
Single-cell sequencing	Cellular composition, transcriptional states, activation/exhaustion profiles	Fresh cells from peripheral blood, BALF, lung tissue	Research sampling according to disease stage; longitudinal sampling is recommended	Several days to weeks	Exploratory molecular signatures; no clinically validated decision thresholds currently available	Characterization of immune heterogeneity at single-cell resolution and identification of immune phenotypes and key regulatory pathways	High-resolution characterization of cellular functional states; enables discovery of immune subsets undetectable by conventional assays	High cost, complex workflow, demanding bioinformatics analysis, and limited applicability for rapid bedside decision- making	Mainly used for mechanistic studies and immune phenotype discovery; standardized clinical workflows have not yet been established
Transcriptomics	Host immune-related gene expression profiles, inflammatory pathway activity, disease-associated gene signatures	Peripheral blood, BALF, tissue samples	Research sampling according to disease stage; longitudinal sampling is recommended	Several days to weeks	Exploratory molecular signatures; no clinically validated decision thresholds currently available	Characterization of host immune response networks; identification of immune biomarkers and prognostic models	Simultaneous assessment of large numbers of immune-related molecular features	Complex data analysis; candidate biomarkers require external validation; variability exists across studies regarding signature reproducibility	Mainly used for biomarker discovery and mechanistic studies
Proteomics	Soluble immune molecules, protein expression profiles	Serum, plasma, BALF	Mainly applied in exploratory studies; serial sampling may be performed in research settings	Several hours to several days	Exploratory biomarkers; no validated clinical thresholds currently available	Identification of immune protein biomarkers with clinical predictive potential	Closely aligned with clinical laboratory testing; some biomarkers have translational potential	Most protein biomarkers lack standardized thresholds and have limited specificity	Some biomarkers have entered clinical research stages, whereas most remain in exploratory and validation phases
Metabolomics	Lipid metabolism, amino acid metabolism, immunometabolites, metabolic pathway alterations	Serum, plasma, BALF	Mainly applied in exploratory studies; serial sampling may be performed in research settings	Several hours to several days	Exploratory biomarkers; no validated clinical thresholds currently available	Characterization of immunometabolic reprogramming and disease severity–associated metabolic alterations	Reflects functional immune status and complements cellular and proteomic information	Strongly influenced by nutritional status, age, and underlying diseases; limited standardization	Mainly used for mechanistic studies and candidate biomarker screening; clinical applicability requires further validation
mNGS (host–pathogen integrated assessment)	Pathogen spectrum, mixed infections, pulmonary microbiome composition, host–pathogen interaction characteristics	BALF, blood, sputum.	Used in cases of diagnostic uncertainty, severe disease, immuno- compromised patients, or suspected mixed infections; selective repeat testing may be considered	24–72 h	Interpretation requires integration of sample quality, pathogen burden, host status, and colonization risk; no immune-related thresholds are applicable	Interpretation requires integration of sample quality, pathogen burden, host status, and colonization risk; no immune-related thresholds are applicable	Broad pathogen coverage; particularly valuable in immunocompromised patients and complex infections	Unable to reliably distinguish colonization from active infection; relatively high cost; requires careful clinical interpretation	Increasingly incorporated into clinical workflows, particularly for difficult-to- diagnose cases and immunocompromised patients

mHLA-DR, Monocyte human leukocyte antigen-DR; BALF, bronchoalveolar lavage fluid; mNGS, metagenomic next-generation sequencing.

### 5.1 Single-cell technologies and immune cell atlas profiling

With the advancement of single-cell technologies, immune assessment in severe pneumonia has progressively expanded from evaluating changes in immune cell abundance to high-resolution characterization of cellular composition, functional states, and cell–cell interactions, providing a novel technological foundation for immune phenotype identification. Emerging evidence indicates that single-cell approaches can uncover critical immune abnormalities that remain undetected by conventional assays. Using single-cell RNA sequencing of peripheral blood immune cells from 100 patients, Xiao et al. (65) demonstrated that patients with severe pneumonia exhibited lymphopenia, increased monocyte proportions, and substantial alterations in T-cell, B-cell, and myeloid cell subsets. Notably, classical monocytes displayed persistent activation of pro-inflammatory pathways, including S100A8/9/12 and TLR4–MYD88 signaling, whereas innate CD8^+^ T-cells exhibited prominent exhaustion characteristics. These findings suggest that immune dysregulation in severe pneumonia is reflected primarily by alterations in cellular functional states rather than simply quantitative abnormalities. Similarly, Zheng et al. (12) further demonstrated in a severe SARS-CoV-2 infection model that CD177-high neutrophils were markedly enriched in lung tissues. They also identified a Stfa-high cell population potentially associated with impaired antigen presentation, providing tissue-level insights into neutrophil-mediated pulmonary immunopathology. Furthermore, a longitudinal study by Zhao et al. (66) in patients with ARDS complicated by hypercapnia showed that the proportions of classical monocytes and inflammation-related molecules, including IL-12p40 and IL-23, changed dynamically throughout disease progression. These temporal alterations were closely associated with clinical deterioration, suggesting that single-cell technologies can further characterize the temporal heterogeneity of immune responses. Collectively, these studies indicate that the greatest advantage of single-cell technologies lies not simply in identifying novel inflammatory cell populations but in reconstructing immune cell composition, functional states, and cellular interaction networks at single-cell resolution, thereby revealing the cellular basis underlying immune heterogeneity among patients. However, current applications of single-cell sequencing remain largely restricted to mechanistic investigations. High costs, complex analytical workflows, prolonged turnaround times, and limited standardization and reproducibility across platforms continue to hinder their direct application for real-time clinical decision-making in intensive care settings (67).

### 5.2 Multi-omics integration and characterization of host response networks

Although single-cell technologies enable detailed characterization of immune cell composition and functional states, they remain primarily focused on cellular-level heterogeneity and cannot fully capture multi-layered alterations involving gene expression, protein function, and metabolic reprogramming during host immune regulation. Therefore, multi-omics approaches, through the integration of information from different biological layers, including transcriptomics, proteomics, and metabolomics, further extend immune monitoring from the cellular level to the level of host-response networks. These approaches may facilitate the identification of key molecular pathways and their interactions that drive the development and progression of severe pneumonia. Transcriptomics enables the identification of novel immune-related genes and the development of prognostic models. For instance, Xu et al. (68) identified IL-1 family– and tumor necrosis factor superfamily–associated response genes in adenovirus type 55 infection, while Armignacco et al. (69) established a 48-gene signature based on whole-blood transcriptomics that can predict early progression from COVID-19 to severe pneumonia. Ma et al. (70)further applied transcriptomic analysis and suggested that the beneficial effects of prostaglandin E1 in severe pneumonia may be associated with suppression of the JAK–STAT signaling pathway, further indicating that transcriptomics can not only identify disease-associated molecular signatures but also facilitate the exploration of potential therapeutic targets. Proteomics, in contrast, focuses more on the discovery of quantifiable biomarkers. Lu et al. (71) identified differentially expressed proteins, including gelsolin, in airway secretions from patients with VAP. Tsuchiya et al. (72) found that soluble CD206, a circulating soluble form of the macrophage mannose receptor expressed by AMs, was closely associated with disease severity and mortality risk in patients with community-acquired pneumonia (CAP), suggesting that proteomics can facilitate the identification of candidate immune biomarkers with clinical translation status. Metabolomics further reveals the link between immunometabolic reprogramming and disease severity. Georgakopoulou et al. (73) demonstrated that alterations in lipid metabolism, particularly the ratio of phosphatidylcholine to lysophosphatidylcholine, are closely associated with the severity of respiratory infections and immune activation. Nguyen et al. (74) found that plasma levels of non-esterified polyunsaturated fatty acids are increased in patients with severe COVID-19 and are associated with fewer ventilator-free days. Kullberg et al. (75) showed that the gut microbiota–derived tryptophan metabolite indole-3-acetic acid exacerbates lung injury by activating the aryl hydrocarbon receptor and enhancing neutrophil reactive oxygen species production. Gjurašin et al. (76) further reported that metabolic dysfunction–associated fatty liver disease shapes a distinct semaphorin–cytokine immune signature in patients with sCAP. These studies collectively indicate that metabolic alterations are not merely accompanying features of inflammatory responses but also represent an important component of host immune regulation. Overall, the major value of multi-omics approaches lies not in replacing conventional immune assays, but in identifying key molecular signatures that reflect host immune status within high-dimensional datasets. By integrating information across multiple biological layers, multi-omics approaches enable a more comprehensive characterization of immune heterogeneity in severe pneumonia and provide a foundation for establishing reproducible and interpretable immune biomarker panels. However, current multi-omics studies still face several challenges, including high costs, complex data integration, lack of standardized analytical workflows, and insufficient external validation. Consequently, their applications remain largely limited to mechanistic investigations and candidate biomarker discovery, and substantial gaps remain before routine clinical implementation can be achieved (77).

### 5.3 Pathogenomics and integrated host–pathogen assessment

Etiological diagnosis represents a critical foundation for precision treatment of severe pneumonia. In recent years, pathogen-oriented omics technologies represented by mNGS have rapidly developed, providing new tools for pathogen identification and the characterization of host–pathogen interactions in complex infectious settings. Unlike approaches such as single-cell profiling and multi-omics integration, which directly assess host immune status, mNGS primarily focuses on the characterization of pathogen profiles, microbial burden, and ecological features of infections. Its clinical value lies in integrating pathogen-related information with host immune backgrounds to enable comprehensive assessment of disease progression, infection patterns, and treatment responses. Multiple studies have demonstrated that mNGS of BALF significantly improves pathogen detection rates, especially in immunocompromised patients and in cases of mixed infections (43, 78, 79). Wang et al. (80) further demonstrated that pathogen profiles are closely associated with host immune status. Reduced CD4^+^ T-cell levels were linked to an increased risk of PJP and complex mixed infections, with a CD4^+^ T-cell count below 100 cells/μL serving as a potential risk indicator in specific immunocompromised populations. However, this threshold was primarily derived from particular immunodeficiency settings, and whether it can be applied to the broader population of patients with severe pneumonia requires further validation. Similarly, Sun et al. (44) reported that the detection rate of opportunistic pathogens increases in parallel with the degree of immunosuppression. Beyond pathogen identification, pathogen-oriented omics technologies have also advanced our understanding of microbiome alterations in severe pneumonia and host–pathogen interactions. Viral reactivation has been suggested to contribute to immune dysregulation in severe pneumonia and has been associated with adverse clinical outcomes. Huang et al. (81) and Liu et al. (82) independently demonstrated that reactivation of CMV, herpes simplex virus type 1, and Epstein–Barr virus in the respiratory tract of patients with sCAP is independently associated with increased mortality. Moreover, Lv et al. (45) identified a remarkably high diversity of rare microbial co-infections in kidney transplant recipients, while Bustos et al. (83) observed temporal shifts in the lung microbiome during the progression of VAP, accompanied by a metabolic transition toward anaerobic pathways. Yang et al. (84) found that pulmonary-to-blood microbial translocation was associated with enhanced systemic inflammatory responses, further highlighting the link between local infection and systemic immune responses. However, detection of pathogen nucleic acids does not necessarily indicate active infection. Particularly in immunocompromised patients, colonization, latent infection, and true invasive infection often overlap, and reliance solely on pathogen detection results may lead to overdiagnosis or inappropriate treatment. Therefore, recent studies have increasingly focused on the clinical interpretation of pathogen detection results. Li et al. (85) developed an mNGS-based prediction model for invasive pulmonary aspergillosis by integrating pathogen-related information with host risk factors, thereby improving the identification of high-risk patients. Similarly, Michel et al. (86) applied 16S rRNA and internal transcribed spacer amplicon sequencing to further improve the discrimination between fungal infection and colonization states. The true value of pathogenomic approaches lies not in replacing conventional microbiological diagnostics but in reinterpreting pathogen-related information within the context of host immune status. Integrating pathogen detection results with host immune profiles enables more accurate identification of distinct infection patterns and immune phenotypes, providing a foundation for subsequent immune phenotype-based stratification and precision intervention strategies.

## 6 Immune phenotype–driven precision stratification

Advances in immune monitoring technologies have enabled precision stratification in severe pneumonia to evolve from traditional clinical scoring systems toward individualized classification based on host immune phenotypes. The primary objective is to identify patient subgroups with distinct prognostic risks, susceptibility to secondary infections, and potential therapeutic response profiles, thereby providing a basis for precision treatment. In recent years, immune stratification strategies have evolved from reliance on single biomarkers toward multiparametric integrated models and machine learning–assisted high-dimensional immune phenotype identification. These approaches have increasingly incorporated host characteristics, pathogen-related information, and dynamic changes in immune responses for integrated assessment.

### 6.1 Biomarker-based immune phenotype identification

Immune biomarkers represent the most direct and widely applied tools for immune phenotype identification in severe pneumonia. Their value extends beyond predicting disease severity, as they provide insights into host immune status and may inform the selection of immunomodulatory strategies (87, 88). Current evidence suggests that patients with severe pneumonia may exhibit distinct immune phenotypic patterns characterized by hyperinflammatory features or immunoparalysis. However, these immune states are not mutually exclusive and may coexist within the same patient, undergoing dynamic changes according to disease stage, pathogen type, and therapeutic interventions (19, 89). (**Table 2**). The immunoparalytic phenotype is mainly characterized by impaired antigen presentation and compromised adaptive immune function. Monocyte human leukocyte antigen-DR (mHLA-DR) is a classical biomarker for assessing innate immune paralysis, and persistently reduced mHLA-DR expression has been strongly associated with increased susceptibility to secondary infections and poor clinical outcomes (90). In addition, decreased CD4^+^ T-cell proportions in BALF and sustained expansion of granulocytic myeloid-derived suppressor cells also indicate suppressed host immune function and are associated with disease severity and mortality risk in pneumonia (91–93). These biomarkers reflect impaired host immune defense capacity from different biological perspectives and may therefore better characterize immunoparalytic states than individual markers alone (94). In contrast, the hyperinflammatory phenotype is characterized by persistent elevation of pro-inflammatory cytokines and excessive activation of innate immune responses. Inflammatory mediators, including IL-6, CXCL10, and granulocyte–macrophage colony-stimulating factor, are closely associated with inflammatory intensity and adverse outcomes in severe pneumonia (40, 95). Moreover, the IL-6/IL-10 ratio provides additional information regarding the dynamic balance between pro-inflammatory and anti-inflammatory responses and has shown potential value for disease severity stratification (96). However, a hyperinflammatory phenotype does not necessarily indicate preserved immune competence. In severe pneumonia, hyperinflammation and immunoparalysis may coexist; therefore, inflammatory biomarkers alone are insufficient to accurately determine whether patients are likely to benefit from immunosuppressive therapies (3, 89). In recent years, several soluble immune molecules have also been investigated for immune stratification. Soluble CD206 and soluble suppression of tumorigenicity 2 (sST2) have been associated with disease severity and mortality risk in patients with CAP and may serve as supplementary indicators of immune activation (97, 98). Soluble programmed cell death protein 1 (sPD-1) and soluble programmed death-ligand 1 (sPD-L1) primarily reflect immune exhaustion and have demonstrated potential prognostic value (99, 100). However, most emerging biomarkers currently lack standardized detection methods and well-defined therapeutic thresholds. Consequently, their clinical applications remain largely limited to risk assessment rather than direct guidance of treatment decisions. Importantly, interpretation of immune biomarkers cannot be separated from host context. Immune phenotype identification should incorporate age, baseline immune status, previous immunosuppressive therapies, and longitudinal disease trajectories for comprehensive assessment. Although immune biomarkers such as mHLA-DR have demonstrated promising clinical potential, evidence remains limited regarding the establishment and validation of population-specific reference thresholds across different host populations. Therefore, current approaches should prioritize integrated interpretation of biomarker dynamics within clinical context rather than the rigid application of universal cutoff values (94).

**Table 2:** Candidate immune phenotypes in severe pneumonia: defining characteristics, representative biomarkers, dynamic features, and potential clinical implications.

Candidate immune phenotype	Biological characteristics	Representative biomarkers	Dynamic features	Potential clinical implications
Hyperinflammatory phenotype	Excessive innate immune activation, cytokine dysregulation, endothelial injury, and coagulation abnormalitie	IL-6, CXCL10, granulocyte–macrophage colony-stimulating factor, NET-related markers, D-dimer, complement components	Predominantly observed during early disease stages but may coexist with immunoparalysis	Risk stratification; identification of patients who may benefit from anti-inflammatory therapeutic strategies in clinical studies
Immunoparalytic phenotype	Impaired monocyte activation, lymphopenia, and T-cell dysfunction	Reduced mHLA-DR expression, decreased CD4^+^/CD8^+^ T-cell ratio, lymphopenia, impaired lymphocyte function	More frequently observed during the persistent or later stages of disease but may also develop early	Monitoring the risk of secondary infections and identifying candidates for investigational immune-enhancing therapies
Mixed phenotype	Concurrent or sequential coexistence of hyperinflammation and immunoparalysis during disease evolution	Integrated biomarker panels, longitudinal biomarker trajectories, and clinical characteristics	Changes dynamically with disease progression, pathogen clearance, and therapeutic interventions	Requires longitudinal multiparametric immune assessment rather than reliance on individual biomarkers

IL-6, Interleukin 6; CXCL10, C-X-C motif chemokine ligand 10; NETs, neutrophil extracellular traps; mHLA-DR, monocyte human leukocyte antigen-DR.

### 6.2 Multiparameter integrated models and risk stratification optimization

Single biomarkers, however, are insufficient to fully capture the complexity of the immune system, and thus multiparameter integrated scoring systems have gradually emerged as a key approach for precision stratification. Traditional tools such as CURB-65, the pneumonia severity index, and the Sequential Organ Failure Assessment score demonstrate limited predictive performance in immunocompromised populations, suggesting that conventional clinical parameters alone are insufficient to fully capture disease heterogeneity (101). Current studies have increasingly explored the integration of immune biomarkers with inflammatory, nutritional, and organ function-related parameters to improve risk prediction performance. For example, Cao et al. (102) established a scoring system for late-onset sCAP in patients following allogeneic hematopoietic stem cell transplantation, integrating monocyte count, albumin, lactate dehydrogenase, and blood urea nitrogen, which effectively stratified 60-day survival risk. Chen et al. (103) reported that a combined score of the systemic immune-inflammation index and the prognostic nutritional index independently correlated with 28-day mortality. It should be noted that although these models incorporate immune-related variables, they essentially remain risk prediction models. The inclusion of immune parameters may improve predictive performance for disease severity, mortality, or adverse outcomes; however, it does not necessarily indicate that biologically distinct subgroups with different immune mechanisms have been identified. Therefore, the current value of most multiparametric models remains primarily in clinical risk stratification rather than true immune phenotype identification. Future models should further integrate biomarkers that directly reflect immune functional states, together with host characteristics, pathogen-related information, and longitudinal immune dynamics, to move beyond risk prediction toward biologically informed immune phenotype stratification and provide a foundation for precision immunomodulatory interventions.

### 6.3 Machine learning–assisted identification of high-dimensional immune phenotypes

With the accumulation of high-dimensional immune datasets, machine learning has increasingly been applied beyond conventional risk prediction toward immune phenotype identification. Unlike traditional multiparametric models that primarily predict mortality or clinical complications, machine learning approaches can integrate immune biomarkers, pathogen-related information, and clinical characteristics to identify patient subgroups with distinct immune features and potential differences in therapeutic responses from complex datasets, thereby providing new stratification strategies for precision immunomodulation. However, current applications of supervised learning methods remain largely focused on risk prediction. Peng et al. (104) applied algorithms such as support vector machines and random forests in kidney transplant recipients to integrate mHLA-DR expression, CD64 index, and absolute lymphocyte subset counts, demonstrating that the model outperformed single biomarkers in identifying pneumonia risk and progression to severe disease. Ma et al. (105) developed a prognostic model for 28-day mortality based on the lactate dehydrogenase-to-albumin ratio, while Li et al. (106) constructed a high-performance predictive model for HAP in patients with traumatic brain injury by integrating age, Glasgow Coma Scale score, computed tomography imaging parameters, and the systemic inflammatory response index. However, these models are primarily designed for clinical risk prediction, and their outputs remain focused on mortality or complication risks rather than the classification of distinct immune states. In contrast, unsupervised learning approaches are more closely aligned with immune phenotype identification. Chen et al. (107) stratified patients with sCAP into two subtypes with significantly different mortality risks based on microbiome features derived from mNGS, and further developed a predictive model incorporating immune status, comorbidities, and specific pathogens. Qin et al. (108) identified three distinct immune phenotypes through unsupervised clustering of immunological parameters, with significant differences observed in inflammatory response intensity and clinical outcomes across these phenotypes. However, current studies mainly rely on immune biomarkers and clinical characteristics for stratification. Whether these identified subgroups possess stable underlying immunobiological mechanisms and whether they can predict differential benefits from immunomodulatory therapies remain to be further validated. Overall, the value of machine learning lies not only in improving predictive accuracy but also in facilitating the transition of severe pneumonia management from clinical risk stratification toward immune phenotype-based stratification.

### 6.4 Current status of clinical translation of immune biomarkers

Immune biomarkers, multiparametric models, and machine learning approaches provide tools for immune stratification in severe pneumonia at different levels; however, their clinical maturity varies considerably (**Table 3**). Currently, peripheral blood cell counts and lymphocyte subset analyses can be used as adjunctive approaches for evaluating host immune status in clinical practice, but their specificity remains limited and these parameters alone are insufficient to determine whether patients exhibit predominantly hyperinflammatory or immunoparalytic immune phenotypes (80, 91, 93). Therefore, the availability of a test in routine clinical practice does not necessarily indicate that the corresponding biomarker is ready to guide precision immunotherapy. Among candidate functional biomarkers, mHLA-DR is one of the most extensively investigated markers of immunoparalysis. Reduced mHLA-DR expression reflects impaired antigen-presenting capacity of monocytes and is associated with secondary infections and adverse outcomes in critically ill patients (90). Standardized flow cytometry can improve inter-laboratory consistency in mHLA-DR measurements; however, the optimal thresholds across different host populations, disease stages, and treatment contexts have not been uniformly validated. Moreover, sufficient evidence demonstrating that mHLA-DR-guided therapeutic adjustment can improve clinical outcomes in severe pneumonia remains lacking (94). Therefore, mHLA-DR is currently more suitable as an adjunctive or research-oriented immune monitoring biomarker in specialized centers rather than an independent tool for routine treatment decision-making. IL-6, IL-10, and the IL-6/IL-10 ratio reflect inflammatory activation and the balance between pro-inflammatory and anti-inflammatory responses and have demonstrated potential value for disease severity assessment and prognostic stratification across different pneumonia cohorts. However, current evidence mainly supports their use as indicators of disease severity and prognosis, while prospectively validated thresholds for immune phenotype classification and biomarker-guided treatment strategies in severe pneumonia remain lacking (40, 95, 96). Therefore, IL-6 should be considered an auxiliary stratification biomarker in the current stage of clinical translation and cannot yet serve as an independent basis for immune phenotype identification or immunotherapy selection in severe pneumonia. Soluble CD206 and sST2 have demonstrated predictive value for disease severity and mortality risk in patients with CAP (72, 97, 98). However, current evidence is mainly derived from observational cohorts, with limited availability of standardized thresholds, sufficient external validation, and biomarker-guided intervention studies. In addition, myeloid-derived suppressor cells and sPD-1/sPD-L1 can reflect the expansion of immunosuppressive cell populations or immune checkpoint-related alterations. Nevertheless, current evidence for these biomarkers in severe pneumonia is primarily derived from specific patient populations or single-center studies, and they remain at an exploratory stage (92, 93, 100).

**Table 3:** Clinical translation status of immune biomarkers in severe pneumonia.

Category	Immune biomarker	Primary immune status reflected	Main clinical application	Clinical translation status	Key limitations
Routine clinical use	Lymphocyte count	Adaptive immune function and the degree of immune deficiency	Adjunctive assessment of host immune status	High	Limited specificity; cannot independently define immune phenotypes or immune-directed interventions
Clinical translation stage	mHLA-DR	Monocyte antigen-presenting capacity and immunoparalysis	Assessment of acquired immunosuppression and evaluation of secondary infection risk	Moderate to high	Standardization, turnaround time, population-specific thresholds, and therapeutic benefit of biomarker-guided intervention remain to be validated
	IL-6	Degree of pro-inflammatory activation	Assessment of inflammatory burden and supportive identification of hyperinflammatory states	Moderate	Elevated inflammation does not necessarily indicate a specific immune phenotype; validated treatment-decision thresholds are lacking
	IL-10 and the IL-6/IL-10 ratio	Balance between pro-inflammatory and anti-inflammatory responses	Assessment of immune dysregulation and prognostic risk	Moderate	Strongly influenced by disease stage; standardized assays and validated cutoff values remain unavailable
Exploratory stage	soluble CD206	Monocyte/macrophage activation state	Adjunctive assessment of disease severity and mortality risk in CAP	Low	Evidence is mainly derived from observational studies, with insufficient external validation for risk prediction in CAP and no established role in treatment guidance
	sST2	Tissue injury-associated inflammatory activation	Prediction of disease severity and adverse outcomes in patients with CAP	Low	Uniform assay standards, validated thresholds, and prospective validation are lacking
	Myeloid-derived suppressor cells	Expansion of immunosuppressive myeloid cell populations	Identification of immunosuppressive states and susceptibility to secondary infection	Low	Technically complex measurement; clinical thresholds and therapeutic implications remain undefined
	sPD-1/sPD-L1	Immune exhaustion and immune checkpoint-related alterations	Assessment of immune exhaustion and exploration of potential immunomodulatory targets	Low	Evidence is largely limited to selected cohorts or single-center studies and is currently insufficient for treatment stratification

Routine clinical use refers to biomarkers already incorporated into clinical assessment workflows. Clinical translation stage refers to biomarkers supported by clinical studies but lacking sufficient validation for treatment guidance. Exploratory stage refers to biomarkers supported mainly by mechanistic or observational studies.

mHLA-DR, Monocyte human leukocyte antigen-DR; IL-6, interleukin-6; IL-10, interleukin-10; CAP, community-acquired pneumonia; sST2, soluble suppression of tumorigenicity 2; sPD-1, soluble programmed cell death protein 1; sPD-L1, soluble programmed death-ligand 1.

## 7 Precision intervention strategies guided by immune monitoring

**Figure 1:**
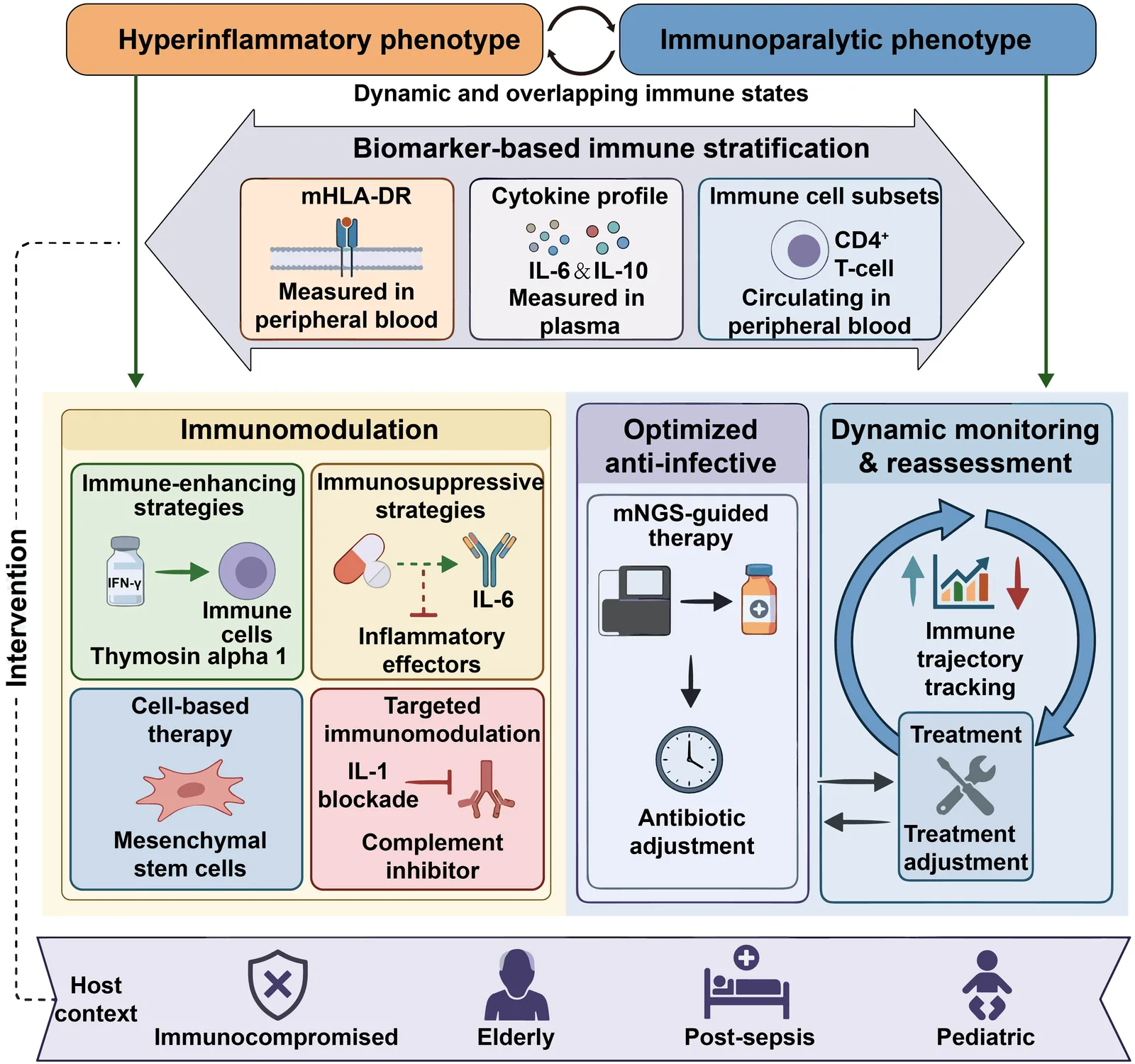
Immune phenotype–guided precision intervention in severe pneumonia. Patients with severe pneumonia may exhibit two major immune phenotypes, including the hyperinflammatory phenotype and immunoparalytic phenotype, which may overlap and dynamically evolve during disease progression. Biomarker-guided immune stratification integrates peripheral blood mHLA-DR expression, cytokine profiles, and immune cell subsets to characterize host immune status and identify potential immune phenotypes. Based on immune profiling, tailored interventions can be considered, including immune-enhancing strategies, immunosuppressive strategies, cell-based therapies, targeted immunomodulation and optimized anti-infective management. Dynamic immune monitoring and reassessment should be implemented throughout the treatment course to track immune trajectories and guide therapeutic adjustments. Host-specific factors, including immunocompromised status, elderly populations, post-sepsis conditions, and pediatric patients, should be incorporated into the evaluation framework to achieve individualized precision management. mHLA-DR, Monocyte human leukocyte antigen-DR; IL-6, interleukin-6; IL-10, interleukin-10.

Advances in understanding the immune heterogeneity of severe pneumonia are contributing to a shift in therapeutic strategies from traditional “one-size-fits-all” approaches toward precision interventions based on immune phenotypes, dynamic immune changes, and potential therapeutic targets (87). The core of this transition is not merely the addition of immunomodulatory measures, but the identification of patients most likely to benefit from specific interventions based on quantitative assessment of host immune status. This approach may support the development of a closed-loop management model encompassing immune enhancement, immunosuppression, cell-based therapies, targeted modulation, and optimized anti-infective treatment (**Figure 1**). It should be noted that not all precision intervention strategies have reached the stage of clinical application. These strategies differ substantially in terms of randomized clinical evidence, definition of target populations, and validation of therapeutic benefits. Therefore, a stratified evaluation of the evidence maturity and clinical translation status of current major intervention strategies is essential for clarifying their practical applicability and future research directions (**Table 4**).

**Table 4:** Evidence maturity and current clinical positioning of precision intervention strategies for severe pneumonia.

Intervention strategy	Evidence maturity	Current clinical positioning	Major limitations
Corticosteroid therapy	High	Supported by relatively robust clinical evidence in selected patients with severe community-acquired pneumonia; application may be considered based on guideline recommendations and individual patient characteristics	Therapeutic benefits may depend on disease phase, pathogen context, and host immune phenotype; treatment decisions cannot be determined by a single biomarker alone
PCT-guided antimicrobial management	Moderate to high	Supported by relatively robust clinical evidence in selected patients with severe community-acquired pneumonia; application may be considered based on guideline recommendations and individual patient characteristics	Cannot replace clinical judgment, pathogen identification, or infection source control
IFN-γ and thymosin α1	Low	Exploratory; should currently be evaluated within clinical research settings	Evidence is mainly derived from small-scale or selected patient populations; optimal beneficiary populations and safety profiles remain uncertain
IL-1/IL-6 targeted blockade	Moderate in COVID-19; low in non-COVID severe pneumonia	Evidence obtained in specific COVID-19 contexts cannot be directly extrapolated to other severe pneumonia populations	Insufficient pathogen-specific evidence, particularly for bacterial sCAP; large-scale validation in immune phenotype-defined populations is lacking
MSC therapy	Low	Exploratory and currently limited to clinical trials	Evidence for improvement in major clinical outcomes remains inconsistent; substantial heterogeneity exists among MSC products and treated populations
Complement-targeted therapy	Low	Primarily at the preclinical and early translational research stages	Evidence is mainly derived from animal models and exploratory studies; clinical endpoint validation is lacking
Bacteriophage therapy	Low	Investigational and mainly restricted to preclinical or early clinical research settings	Current evidence relies largely on case reports and animal studies; target populations and standardized treatment protocols remain undefined
Nanomaterial-mediated immunomodulation	Very low	Currently limited to the preclinical research stage	Human safety and efficacy data are lacking

PCT, Procalcitonin; IFN-γ, interferon-gamma; IL-6, interleukin 6; IL-10, interleukin 10; IL-1, interleukin 1; COVID-19, coronavirus disease 2019; sCAP, severe community-acquired pneumonia; MSC, mesenchymal stem cell .

### 7.1 Indications for immune-enhancing therapy

In the context of immune-enhancing strategies, the key lies in the accurate identification of acquired immunosuppression (88). Persistently reduced expression of mHLA-DR is an important marker of immunoparalysis following sepsis and severe pneumonia, and provides a theoretical basis for the use of immunostimulatory agents such as interferon-gamma (IFN-γ) (109). Grimm et al. (90) reported that in a patient with COVID-19-related ARDS complicated by multidrug-resistant bloodstream infection, administration of IFN-γ in the setting of markedly reduced mHLA-DR expression significantly restored its levels and was accompanied by improved infection control. However, a phase II randomized controlled trial conducted by Roquilly et al. (110) for the prevention of HAP was terminated early due to a trend toward potential harm. The study also found that patients with lower CCL17 responses after treatment were more likely to develop nosocomial infections. Therefore, IFN-γ cannot currently be considered a routine immune-enhancing therapy for severe pneumonia and should only be further evaluated in carefully selected patients within clinical research frameworks. Future studies should incorporate biomarkers to identify patient populations that are most likely to benefit from such therapy. In contrast, thymosin alpha 1 has demonstrated potential immunomodulatory effects and signals of clinical benefit in studies of COVID-19 and sepsis; however, the available evidence remains inconsistent. Systematic reviews of COVID-19 have reported conflicting conclusions regarding major clinical outcomes, including mortality (111, 112). Moreover, a recent randomized controlled trial in adults with sepsis failed to demonstrate a significant reduction in 28-day mortality with thymosin alpha 1 treatment (113). Therefore, thymosin alpha 1 cannot currently be recommended as a routine immune-enhancing therapy for severe pneumonia, particularly given the lack of high-quality randomized evidence in non-COVID severe pneumonia populations and in patients selected according to immune phenotypes.

### 7.2 Stratified application of immunosuppressive therapy

The primary goal of immunosuppressive therapies is to mitigate pulmonary and organ injury caused by excessive host inflammatory responses. Among currently available strategies, corticosteroids have the most robust supporting evidence; however, their individualized implementation must rely on dynamic immune assessment. Dequin et al. (114), in a large randomized trial, demonstrated that hydrocortisone reduced 28-day mortality and the need for tracheal intubation in patients with sCAP. Furthermore, the 2024 updated guidelines from the Society of Critical Care Medicine issued a strong recommendation for the use of corticosteroids in hospitalized adults with bacterial sCAP (115). Therefore, for adult patients meeting the criteria for bacterial sCAP without clear contraindications, corticosteroid therapy is supported by sufficient evidence for clinical implementation. However, the efficacy of corticosteroids cannot be simply extrapolated to all treatment regimens or all types of pneumonia. Meduri et al. (116) found that initiating prolonged low-dose methylprednisolone therapy 72–96 hours after admission did not significantly reduce 60-day mortality in patients with sCAP, suggesting that treatment effects may be influenced by differences in corticosteroid type, timing of initiation, treatment duration, and patient selection. In addition, corticosteroids may exacerbate secondary immunosuppression. Cour et al. (117) observed that COVID-19 patients receiving dexamethasone treatment exhibited significantly reduced mHLA-DR expression and CD4^+^ T-cell counts, accompanied by earlier onset of VAP. Pasqua et al. (118) further emphasized that widespread corticosteroid use requires attention to the development of a secondary immunosuppressive window. These findings indicate that treatment decisions should incorporate dynamic assessment of pathogen control, risk of secondary infection, and immune status, rather than relying solely on individual inflammatory biomarkers to determine treatment initiation or prolongation. In addition, targeted blockade of key pro-inflammatory cytokines, such as IL-6, has increasingly attracted attention. IL-6-targeted therapies should be considered separately from corticosteroid treatment. Tocilizumab has demonstrated clinical benefits in several randomized trials of severe COVID-19. Evidence suggests that in hospitalized or critically ill patients with hypoxia and systemic inflammation, who are typically receiving concomitant corticosteroid therapy, IL-6 receptor blockade may reduce mortality or improve organ support outcomes (119, 120). However, these findings are highly dependent on the specific pathological context of COVID-19, and current evidence remains insufficient to support the routine use of IL-6 blockade in non-COVID-19 severe pneumonia.

### 7.3 Cell-based therapy–mediated immune remodeling strategies

Cell-based therapy, particularly mesenchymal stem cell (MSC) therapy, provides an alternative pathway for precision immunomodulation (121). MSCs can interact with T-cells, B-cells, and macrophages while secreting various cytokines, thereby suppressing Th1/Th17 inflammatory responses and promoting regulatory T-cell generation. This dual capacity to inhibit excessive inflammation and facilitate tissue repair confers unique advantages, making MSC therapy an important adjunct in immunomodulatory treatment for severe pneumonia (122, 123). Currently, studies on MSC therapy for severe pneumonia have mainly focused on COVID-19-associated pneumonia. Early clinical studies have suggested that MSC infusion has a favorable safety profile and may improve inflammatory markers and pulmonary function. Shetty (124) reported preliminary safety and potential benefit of MSC infusion in patients with severe COVID-19 pneumonia, and Huang et al. (125) observed successful outcomes in complex cases. However, existing studies indicate variable efficacy, suggesting that host immune background may determine therapeutic response. In recent years, randomized controlled trials have further evaluated the actual therapeutic efficacy of MSC therapy in severe pneumonia. Laterre et al. (126) conducted a randomized, double-blind, placebo-controlled trial to assess the safety and efficacy of allogeneic adipose-derived MSC therapy in patients with sCAP. The results showed that MSC treatment was generally safe but did not significantly improve major clinical outcomes, including clinical cure rates and organ function recovery. Further analysis of host responses demonstrated that MSCs could modulate certain inflammation-related pathways and immune responses; however, these immunomodulatory effects have not yet translated into clear clinical benefits (127). These findings suggest that MSC therapy may exert biological activity, but the ability to regulate immune responses does not necessarily translate into improved patient outcomes. Notably, the efficacy of MSC therapy may be influenced by host immune status. Liao et al. (128) found that IL-18-preconditioned human umbilical cord MSC exhibited enhanced immunomodulatory capacity in a viral pneumonia model and increased T-cell suppressive activity, suggesting that MSC functional optimization and immune phenotype-guided patient selection may represent important strategies for improving therapeutic efficacy in the future. However, clinical studies have not yet demonstrated that specific immune phenotypes can predict responses to MSC therapy, and prospective evidence supporting the selection of MSC-responsive patients based on immune biomarkers remains lacking.

### 7.4 Precision modulation of targeted immune pathways

Targeted immunomodulatory strategies directly act on specific inflammatory pathways or pathogenic processes. For patients with a well-defined cytokine storm, targeting IL-1 has emerged as a representative therapeutic strategy. This approach is gradually expanding from COVID-19-associated pneumonia to other severe infections and pneumonia settings; however, its clinical benefits remain highly dependent on patient selection. In COVID-19-related studies, the SAVE-MORE trial demonstrated that selecting patients based on host inflammatory status could lead to clinical benefits (129). More recently, the INSPIRE randomized trial in patients with non-COVID pneumonia provided preliminary evidence that IL-1 blockade may reduce the risk of pneumonia progression (130). However, current studies remain limited in sample size, and large-scale validation across different pathogen types and immune phenotypes of severe pneumonia patients is still lacking. Therefore, IL-1 blockade should currently be considered a candidate precision intervention guided by immune phenotyping rather than a broadly applicable therapeutic strategy. Beyond this, the complement system and inflammatory signaling pathways have also emerged as important therapeutic targets. Meng et al. (131) proposed the use of advanced materials to “capture” cytokines and mitigate cytokine storm, while Ci et al. (132) developed an immunosuppressive dead-cell system with both lung-targeted delivery and cytokine adsorption capabilities. At the level of classical inflammatory pathways, inhibitors such as *Tetrastigma hemsleyanum* polysaccharides (133) and berberine (134) have demonstrated potential in preclinical studies to alleviate severe pneumonia by modulating Toll-like receptor 4/nuclear factor-κB signaling, immunometabolism, and apoptotic processes. However, these strategies are currently supported mainly by animal experiments or *in vitro* studies, and their safety, dose optimization, and clinical efficacy remain unvalidated. Therefore, they cannot yet be considered clinical treatment options.

### 7.5 Integration of immune quantification in anti-infective therapy

As a cornerstone of severe pneumonia management, anti-infective therapy is increasingly being integrated into immune monitoring–guided frameworks. The most clinically mature application currently involves optimizing antimicrobial management based on infection-related indicators. Procalcitonin (PCT)-guided adjustment of antibiotic duration has been supported by relatively robust clinical evidence. Studies have shown that PCT-guided discontinuation strategies can reduce antibiotic exposure without increasing the risk of mortality (135). In addition, mNGS has gradually been incorporated into the clinical diagnostic and management workflow of severe pneumonia. Current studies suggest that early antibiotic optimization guided by mNGS findings is associated with improved clinical outcomes in ICU patients with sCAP, with more pronounced benefits observed among immunocompromised patients (136). Therefore, mNGS results may facilitate pathogen identification and antimicrobial regimen adjustment. However, current evidence supporting its clinical benefits is primarily derived from retrospective cohorts and observational studies, and its effects on patient survival, antibiotic exposure, and long-term outcomes require further validation in prospective studies. In the context of multidrug-resistant infections, Duplessis et al. (137) and Li et al. (138) demonstrated the potential of bacteriophage–antibiotic combination therapy and nanocarriers targeting metallo-β-lactamases, respectively, providing novel precision strategies for pathogen eradication in scenarios where conventional antibiotics are ineffective. However, these strategies currently lack sufficient clinical validation and remain at an exploratory stage.

## 8 Challenges and future perspectives

Despite the significant opportunities that immune monitoring may offer for precision management of severe pneumonia, its clinical translation still faces multiple challenges. First, current immune assessment technologies lack standardized protocols, and substantial variability exists across different platforms and methodologies, limiting data comparability and the feasibility of large-scale validation studies. Second, although multi-omics and single-cell approaches have identified numerous potential biomarkers, their translation into rapid, bedside-applicable tools remains challenging, and their clinical accessibility and cost-effectiveness require further evaluation (139). Moreover, effective integration between pathogen identification and immune status assessment has yet to be achieved. This is particularly evident in immunocompromised patients, where uncertainties persist regarding the differentiation between colonization and true infection, the identification of mixed infections, and the clinical significance of viral reactivation (140–142). Collectively, these limitations hinder the full incorporation of immune-guided strategies into routine clinical practice.

Building on this foundation, future research is gradually shifting from “mechanistic elucidation” toward “enhancing clinical utility.” On one hand, the development of rapid, standardized bedside immune function assays may represent a critical step toward achieving precision management (88). These tools should ideally dynamically reflect key immune parameters to support real-time decision-making (143). On the other hand, the integration of multi-omics data with artificial intelligence methods holds promise for constructing high-dimensional predictive models that combine clinical information, immune features, and pathogen data, enabling more accurate identification of high-risk populations and prediction of treatment responses (3). Simultaneously, deeper investigation into the immune landscapes of special populations will further inform individualized strategies, allowing therapeutic regimens to better align with host-specific differences (144, 145).

From a longer-term perspective, the management of severe pneumonia is expected to evolve toward a biomarker-centered dynamic decision-making framework, in which integrated immune and pathogen stratification is performed early in the disease course, followed by real-time adjustment of therapeutic strategies through continuous monitoring. Recent clinical studies guided by immune phenotypes have provided preliminary evidence potential clinical benefits of this approach. Future efforts should further extend this strategy to CAP, VAP, and their specific subpopulations, and apply it to identify patients most likely to benefit from corticosteroids (146), macrolides (147, 148), intravenous immunoglobulin, and other emerging immunomodulatory agents (149). It should be noted that current evidence for some immune phenotypes and therapeutic strategies is derived primarily from COVID-19-related studies, and their applicability to severe pneumonia caused by other pathogens, particularly bacterial sCAP, requires further validation. Therefore, future studies should integrate different pathogen types, host backgrounds, and disease stages to establish more broadly applicable immune stratification systems. At the same time, with the support of rapid molecular diagnostics and dynamic biomarkers, anti-infective therapy may become increasingly precise, thereby improving therapeutic efficacy while reducing the risk of antimicrobial resistance (150). Overall, precision management driven by immune monitoring is gradually transitioning from concept to clinical practice, and its full potential will depend on continued advances in technological standardization and the accumulation of high-quality clinical evidence.

## 9 Conclusions

Overall, the management of severe pneumonia is transitioning from a traditional pathogen-centered empirical treatment paradigm toward a precision medicine framework grounded in host immune status. Immune monitoring not only provides a critical perspective for understanding disease heterogeneity but also provides a foundation for patient stratification and individualized interventions. With the advancement of high-dimensional analytical technologies and strengthened multidisciplinary collaboration, a closed-loop management model encompassing “immune assessment–precision intervention–dynamic monitoring” may be developed, enabling continuous optimization of therapeutic strategies. Although challenges such as the lack of standardization and limited clinical evidence remain, ongoing research efforts are likely to advance the field. Immune-guided precision therapy represents a promising future direction in the management of severe pneumonia. However, its integration into routine clinical practice will depend on several key requirements, including standardized diagnostic methods, immune phenotyping approaches that incorporate pathogen characteristics and disease phases, externally validated predictive models, and adequately powered prospective clinical trials. These trials should determine whether biomarker-guided treatment provides benefits beyond guideline-based standard management and improves clinically meaningful patient outcomes.
